# Functional Classification of Tropical Cattle Feeding Systems Reveals Consistent Associations with Dairy Lipid Quality, Enteric Methane Emissions, and Preclinical Metabolic Outcomes: A Multi-Study Narrative Synthesis

**DOI:** 10.3390/vetsci13090988

**Published:** 2026-09-18

**Authors:** Mario Cuchillo-Hilario, Margarita Díaz-Martínez, Gustavo Flores-Coello, Claudia Delgadillo-Puga, José Nahed-Toral

**Affiliations:** 1 Departamento de Nutrición Animal, Instituto Nacional de Ciencias Médicas y Nutrición Salvador Zubirán (INCMNSZ), Tlalpan 14080, Ciudad de Mexico, Mexico; margarita.diazm@incmnsz.mx (M.D.-M.); claudia.delgadillop@incmnsz.mx (C.D.-P.); 2 Departamento de Ciencias Biológicas, Facultad de Estudios Superiores de Cuautitlán, Universidad Nacional Autónoma de México, Cuautitlán Izcalli 54714, Estado de Mexico, Mexico; 3 Facultad de Medicina Veterinaria y Zootecnia, Universidad Nacional Autónoma de México, Coyoacán 04510, Ciudad de Mexico, Mexico; gfc15sheep@gmail.com; 4 Departamento de Agricultura, Sociedad y Ambiente, El Colegio de la Frontera Sur (ECOSUR), San Cristóbal de Las Casas 29290, Chiapas, Mexico; jnahed@ecosur.mx

**Keywords:** biodiverse grazing, silvopastoral, methane emissions, one health, metabolic health

## Abstract

Administrative labels such as “organic” or “pasture-fed” animal food products inadequately capture the biological complexity of dairy production systems and consequently fail to differentiate potentially meaningful associations with nutritional quality, environmental impact and consumers’ health. We developed a functional classification framework based on three measurable traits: (1) botanical diversity, (2) concentrate dependence, and (3) input intensity, and applied it to six observational and/or experimental studies from tropical Mexico (n = 109 farms across Yucatán, Chiapas, and Colima federal states). Animal production systems were categorized as: (a) biodiverse grazing (silvopastoral, organic, or traditional grazing; >15 plant species or 8–14 species with concentrate < 25% dry matter intake and no synthetic inputs) or (b) conventional (monoculture/indoor systems; <5 plant species or 6–10 species with concentrate >40% with a use of agrochemicals). Biodiverse grazing systems produced lower daily milk yields (6.1 vs. 7.7 L d^−1^; *p* < 0.0001) but yielded milk with more favorable cardiovascular lipid indices: atherogenic index (AI: 1.6–2.2 vs. 1.9–2.5), thrombogenic index (TI: 2.3–2.7 vs. 2.3–3.1), and health promotion index (HPI: 0.46–0.67 vs. 0.40–0.54). Artisan cheese from biodiverse systems contained substantially higher concentrations of tocopherols (127 vs. 77 mg 100 g^−1^ DM) and monoterpenes (460–475 vs. 111–126 ng kg^−1^). An intensive silvopastoral system reduced enteric methane emissions by 18.2% (376 vs. 460 g d^−1^; *p* < 0.05) and by 21.3% per unit dry matter intake (24.0 vs. 30.5 g kg^−1^ DMI; *p* < 0.0001). Forage nutritional quality (crude protein, neutral detergent fiber, acid detergent fiber) varied by species and season, with these parameters influencing estimated methane emissions. In a C57BL/6 murine model of high-fat diet-induced obesity, milk from biodiverse silvopastoral systems attenuated body weight gain (30.5 vs. 36.8 g; *p* < 0.05), preserved glucose tolerance (AUC: 32,616 vs. 45,248), nearly eliminated hepatic macrovesicular steatosis, and promoted smaller adipocyte size (1863 vs. 3880 µm^2^; *p* < 0.05) compared with conventional milk. These findings suggest that functional ecological traits provide a useful framework for evaluating dairy systems, with biodiverse grazing offering a potential convergence of environmental sustainability and preclinical health benefits. Nevertheless, external validation across diverse tropical regions and direct human intervention trials remain necessary to establish generalizability, and the associations identified should be considered hypothesis-generating rather than confirmatory.

## Simple Summary

Consumer demand for dairy products from pasture-based or organic systems has grown substantially. Yet administrative labels such as “organic” or “pasture-fed” food commodities inconsistently predict nutritional quality or environmental impact across production contexts. This narrative synthesis integrates six original studies from tropical Mexico using a functional classification grounded in three core measurable traits: (1) botanical diversity of grazing areas, (2) concentrate dependence, and (3) synthetic input intensity. Biodiverse grazing systems were defined as those with ≥15 plant species, <25% concentrate present in the animal diet, and no use of agrochemical inputs. Within the included studies, this system was associated with milk and cheese with more favorable fatty acid profiles, approximately doubled tocopherol concentrations, and markedly higher terpene content compared with conventional monoculture systems. Similarly, enteric methane emissions were 18% lower per animal and 21% lower per unit of dry matter intake in biodiverse systems. In a murine model of diet-induced obesity, milk from biodiverse systems attenuated body weight gain, preserved glucose tolerance, completely prevented hepatic steatosis, and promoted healthier subcutaneous adipose tissue remodeling relative to conventional milk. Nevertheless, these findings represent merely associations and preclinical evidence; external validation and further human intervention trials are required before causal claims or consumer health protection can be established.

## 1. Introduction

Milk’s nutritional value is not an intrinsic, fixed property but rather arises from a complex interaction of forage ecology, animal nutrition, rumen microbial metabolism, animal metabolism and mammary gland physiology. Over the past two decades, a substantial body of work has established that forage-based diets increase milk concentrations of metabolites and beneficial fatty acids such as α-linolenic acid (ALA, C18:3 n-3), vaccenic acid, and conjugated linoleic acid (CLA) isomers compared with total mixed rations [1]. This foundational understanding has been expanded by studies demonstrating that forage systems are key drivers of milk composition specificity across contrasting production systems, even in challenging environments such as mountain pastures [2]. It is accepted that forage-based rations can enhance the fatty acid profile of milk sufficiently to be detectable through different diet models’ outcomes [3]. The transfer of plant secondary metabolites from forage to milk has been documented in both bovine and caprine systems. Likewise, Cuchillo et al. [4] demonstrated that goat milk cheeses from animals grazing on Mexican rangelands exhibited significant antioxidant activity, attributed to bioactive polyphenols present in the forage species consumed. This observation aligns with our findings of enhanced tocopherol and terpene concentrations in cheeses from biodiverse grazing systems in bovines.

In tropical regions such as Chiapas, Mexico, this potential is further amplified. Livestock production in these areas increasingly integrates trees, shrubs, and grasses, collectively termed silvopastoral systems, which mitigate enteric methane emissions while maintaining or improving animal productivity [5,6,7,8]. Recent evidence suggests that these biodiverse systems influence milk quality, improving atherogenic (AI) and thrombogenic (TI) indices when compared with conventional models [9,10]. Furthermore, metabolomic relationships between grazing and milk composition are being investigated, demonstrating that specific plant secondary metabolites can resist ruminal metabolism and be transferred to milk, and derived products potentially influencing consumer health [11,12,13]. Therefore, the transfer of bioactive polyphenols from forage to dairy products has been particularly well-documented in caprine systems, where grazing on diverse rangelands significantly enhanced the antioxidant capacity of milk and cheese [4,14].

Despite these advances, the field suffers from a persistent and consequential limitation: the use of administrative labels as proxies for biological performance. Terms such as “organic,” “conventional,” “pasture-fed,” and “grass-fed” are widely employed by certifiers, marketers, and consumers, yet they are surprisingly poor predictors of nutritional quality or environmental impact [15,16]. For instance, a certified organic farm may operate a monoculture pasture with minimal plant diversity using only allowed inputs, while a non-certified silvopastoral system may support > 15 plant species and outperform the organic system on multiple quality metrics. Similarly, “pasture-fed” refers to what animals eat but not which plants they consume; a monoculture of *Cynodon* spp. differs fundamentally from a multi-species sward containing legumes, forbs, and trees.

This imprecision carries real implications. Policy recommendations based on administrative labels may prove inconsistent, and meta-analyses that pool studies under these labels risk confusing true causal relationships. Moreover, the vast majority of mechanistic studies have been conducted in temperate regions, leaving tropical production systems severely understudied. Tropical forages such as *Cynodon* spp., *Megathyrsus maximus*, and *Leucaena leucocephala* contain different concentrations of condensed tannins, saponins, and lignin, which alter ruminal biohydrogenation pathways in ways lacking direct temperate analogues [17,18]. Therefore, direct extrapolation from temperate data is unreliable. Also, existing studies rarely integrate environmental and nutritional outcomes within the same experimental framework. Methane mitigation research typically stops at emissions measurement without examining production or product quality [5], while dairy composition studies rarely consider enteric emissions or animal welfare assessments. The analysis fragmentation performed on different stages in the production chain complicates the complete analysis of potential trade-offs and synergies.

The need for biologically informative classification systems has been increasingly recognized in the recent literature. Musati et al. [19] demonstrated that the botanical biodiversity of pastures modifies ruminal and faecal microbiota, digestion processes, and milk composition in dairy cows. Similarly, Dwan et al. [20] showed that increasing sward species diversity affects enteric methane emissions from dairy cows in rotational grazing systems, though the direction and magnitude of these effects depend on breed and management context. Likewise, recent advancements in milk metabolomics have allowed researchers to discriminate milk origin based on metabolite assessment [21,22]. Also, some other studies emphasized that connecting grazing and milk metabolomes can enhance consumer health by identifying bioactive compounds that survive digestion [12]. In parallel, studies of multispecies swards have demonstrated consistent improvements in milk fatty acid profiles and oxidative stability of the milk matrix [23,24]. Furthermore, the integration of One Health principles into ruminant production research has highlighted the need for holistic assessment frameworks that simultaneously consider animal productivity, animal wellbeing, environmental sustainability, food animal quality and human health outcomes [25].

To address these gaps, we propose a functional classification system based on three measurable traits: (i) botanical diversity, (ii) concentrate dependence, and (iii) input intensity. We then re-analyzed six independent studies from our research group conducted in tropical Mexico during the last two decades, classifying each treatment into one of two categories: (a) biodiverse grazing systems (silvopastoral, organic, or traditional grazing) or (b) conventional systems (monoculture/indoor, low botanical diversity, and high input dependence) [26,27]. We hypothesized that systems characterized by higher forage diversity, lower concentrate dependence, and lower synthetic input intensity would be associated with: (i) improved dairy lipid quality; (ii) enhanced antioxidant transfer; (iii) reduced methane emissions; and (iv) improved preclinical metabolic outcomes in a murine model of diet-induced obesity, compared to low-botanical diversity or high-input systems.

The present study contributes to the literature by providing a systematic narrative synthesis that integrates diverse outcome dimensions: nutritional, environmental, and preclinical metabolism, within a unified functional classification framework, applied specifically to tropical production systems. Because the classification thresholds were derived from the included studies and were not formally tested against alternative values, this narrative synthesis should be read as an exploratory framework rather than an empirically validated classification. It nevertheless illustrates the potential utility of moving beyond administrative labels toward biologically meaningful categories that reflect measurable ecological and management characteristics.

## 2. Materials and Methods

### 2.1. Study Selection and Classification Criteria

Six primary studies were selected from our research group’s work conducted between 2004 and 2024 based on the following eligibility criteria: (a) evaluating cattle production systems in tropical sub-humid Mexico; (b) reporting at least one of the following outcome measures: milk or cheese fatty acid profiles, health-related lipid indices (atherogenic index, AI; thrombogenic index, TI; health promotion index, HPI), tocopherols, terpenes, enteric methane emissions, forage nutritional quality, or preclinical metabolic outcomes in a C57BL/6 murine model; and (c) providing sufficient detail on botanical forage composition, concentrate supplementation, and input management to allow objective functional classification. The formal inclusion criteria encompassed: peer-reviewed articles and academic theses; original data on dairy cattle (crossbred *Bos taurus* × *Bos indicus*) in tropical Mexico; and reporting of at least one outcome of interest with sufficient management detail to assign a functional category.

**Figure 1:**
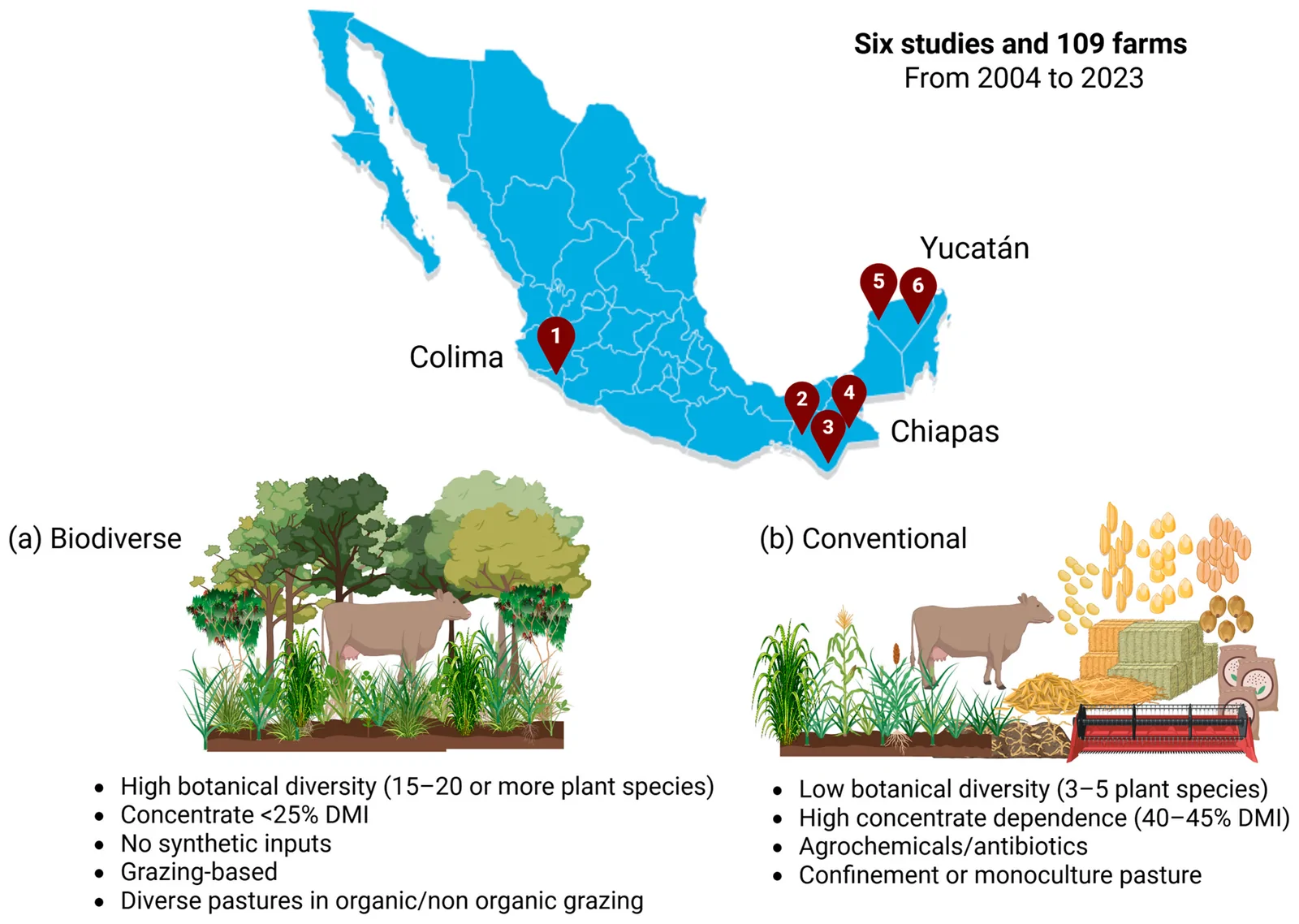
Study sites in tropical Mexico: (**a**) biodiverse grazing systems (silvopastoral, organic, and/or traditional grazing) and (**b**) conventional feeding systems (low botanical diversity, high inputs, monoculture/indoor systems) for cattle production systems in tropical Mexico. DMI = Dry matter intake. 1 = Galina et al. [28]; 2 = Cruz-Morales [10]; 3 = Delgadillo-Puga et al. [9]; 4 = Marroquín-Aguilar [29]; 5 = Flores-Coello et al. [5]; and 6 = Cuchillo-Hilario et al. [30].

Although we did not conduct a systematic literature review for further independent studies, our selection was restricted to studies from our own research group to ensure consistent application of the functional classification criteria and access to detailed data. This approach introduces a risk of selection bias, which we acknowledge as a limitation. The six eligible studies included in this narrative review were: Galina et al. [28]; Cruz-Morales [10]; Delgadillo-Puga et al. [9]; Marroquín-Aguilar [29]; Flores-Coello et al. [5]; and Cuchillo-Hilario et al. [30]. The selected investigations were conducted at three distinct sites in Mexico, each comparing biodiverse and conventional cattle feeding systems (Figure 1). Table S1 (Supplementary Materials) provides detailed characteristics of each source study, including years of data collection, locations, breeds, study designs, and specific outcomes evaluated.

### 2.2. Functional Classification of Cattle Feeding Systems

**Figure 2:**
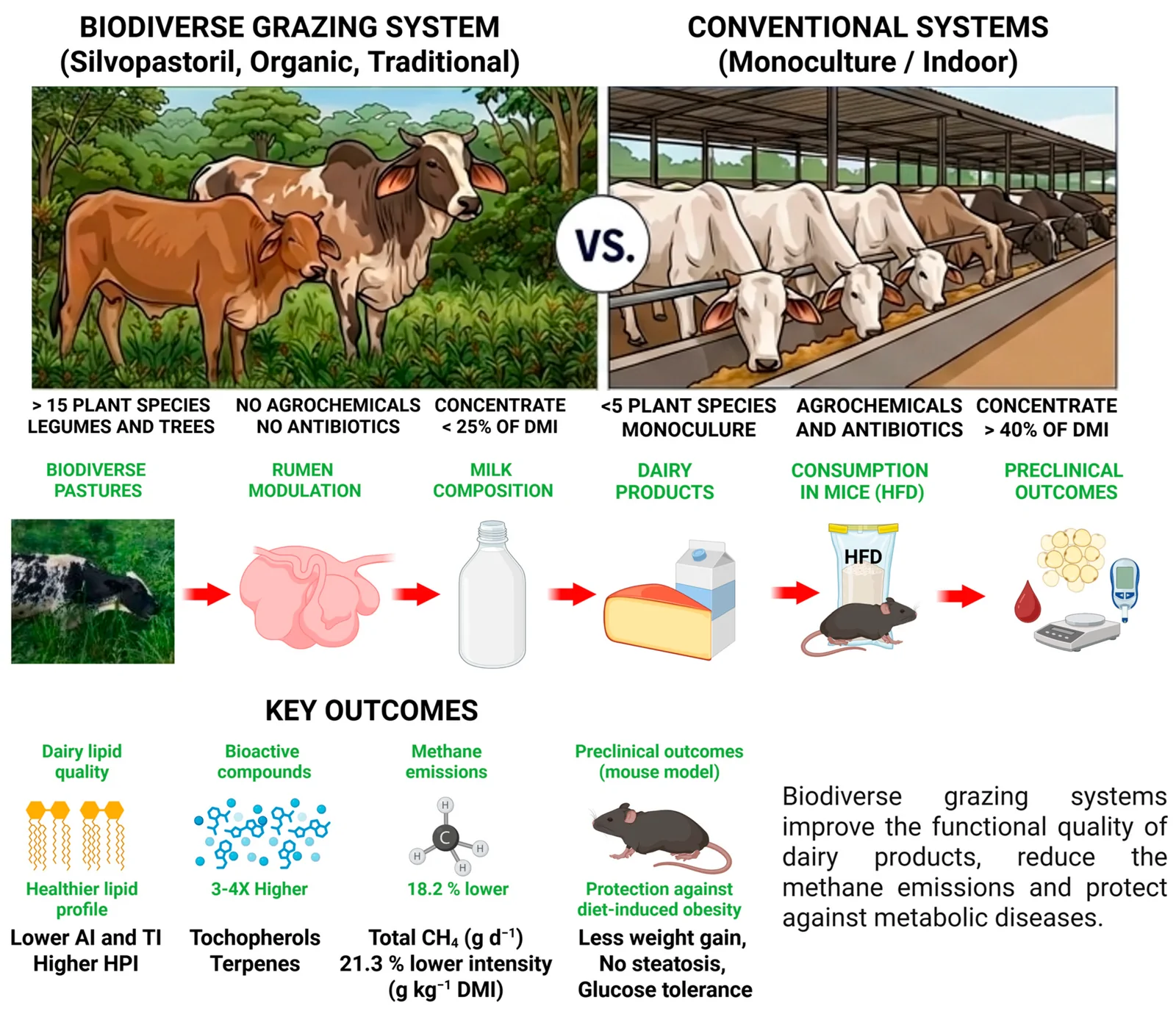
From pasture botanical diversity to metabolic health through biodiverse grazing systems. This figure presents a conceptual framework illustrating the hypothesized relationships between functional system characteristics and observed outcomes. The arrows represent biological associations supported by the synthesized evidence but do not imply direct causal links that were experimentally demonstrated within a single integrated design. CH_4_ = methane; DM = dry matter; HFD = high-fat diet; AI = Atherogenic Index; TI = Thrombogenic Index; HPI = Health Promotion Index.

To move beyond administrative labels, systems were classified as:

1. Biodiverse Grazing if they met at least one of two biological conditions: (i) a diet consisting of >15 plant species, or (ii) the presence of 8–14 species combined with concentrate dependence < 25% of total dry matter intake (DMI) and no routine use of synthetic inputs.
2. Conventional (including monoculture or indoor-based models): (i) if it was characterized by <5 plant species, (ii) or 6–10 species supplemented with a concentrate dependence > 40%, or the routine use of agrochemicals and antibiotics (Figure 2).

The numerical thresholds for botanical diversity (≥15 species, 8–14 species, <5 species) were derived from the distribution of plant species richness observed across the included studies and from ecological literature indicating that functional diversity benefits typically plateau beyond approximately 15 species in tropical pasture systems. The concentrate thresholds (<25% and >40% of DMI) reflect the range commonly used to distinguish low- input systems versus high-input systems in tropical dairy production [26,27]. We acknowledge that these thresholds require empirical calibration through sensitivity analyses, which we outline as a priority for future research. Based on this classification, cattle production systems in tropical Mexico were grouped into two distinct functional categories, as summarized in Table 1.

### 2.3. Data Collection and Synthesis

Data items collected from the selected studies included means and standard errors for: (i) fatty acid profiles (SFA, MUFA, PUFA, and individual long-chain FAs); (ii) lipid health indices (AI, TI, and HPI); (iii) bioactive compounds (tocopherols and terpenes); (iv) enteric methane emissions; (v) animal performance; (vi) forage nutritional quality; and (vii) preclinical metabolic outcomes in C57BL/6 mice. Ranges reported in tables represent the dispersion across different studies or experimental seasons within each functional category.

The values presented in Table 2, Table 3 and Table 4 were reproduced directly from the cited source publications, with ranges calculated from the reported means and standard errors across treatments within each functional category. No new primary data were generated in the present synthesis. Methane estimation methodologies were integrated from two approaches: direct on-farm measurements via sniffer technology (Yucatán; [5]) and mathematical modeling using IPCC Tier 1 and Level 2 equations (Chiapas; [29]). We explicitly distinguish these two types of evidence throughout the Results and Discussion, as they differ fundamentally in uncertainty and methodological basis. The latter estimated emissions based on gross energy (GE) intake and net energy requirements for maintenance (ENm), activity (ENa), and lactation (ENl) tailored to the local herd’s physiological profiles.

### 2.4. Narrative Synthesis and Heterogeneity

A formal meta-analysis was considered inappropriate due to substantial methodological and biological heterogeneity across the data sources, including variations in study design (observational vs. longitudinal/experimental), cattle breed diversity (Gyr × Holstein, Brown Swiss × Brahman, Zebu), differences in analytical platforms (varying gas chromatography protocols), and the distinct nature of comparator systems (indoor vs. grazing, organic vs. conventional). Consequently, a narrative synthesis approach was employed, supported by systematic tabulation and qualitative comparison of trends across functional categories [31].

The narrative synthesis followed the framework of Popay et al. [31] comprising: (1) preliminary synthesis through tabulation and grouping of study characteristics; (2) exploration of relationships within and between studies; and (3) assessment of the internal consistency of the synthesis through qualitative comparison of directional trends. We did not conduct a formal quality assessment of individual studies, as all originated from our research group and employed consistent methodological standards appropriate to their respective designs (observational, cross-sectional, and experimental).

### 2.5. Reliability of Functional Classification

The internal consistency of the functional classification was appraised by comparing the direction of outcomes between treatments assigned to the “Biodiverse” and “Conventional” categories within each included study. In all instances, the direction of effect, wherein biodiverse grazing systems demonstrated superior outcomes for AI, TI, HPI, antioxidant transfer, and methane mitigation, was consistent. While these patterns were internally consistent within the included studies, we acknowledge that this re-analysis identifies recurring associations rather than definitive causal determinants. Therefore, independent external validation across diverse agroecological contexts remains necessary. We recognize that formal sensitivity analyses were not performed to validate the classification thresholds. The selected thresholds were derived from the distribution of plant species richness observed across the included studies, but they were not tested against alternative threshold values. Future work should systematically evaluate alternative threshold values using larger and more diverse datasets.

## 3. Results

### 3.1. Milk Fatty Acid Profiles and Health-Related Lipid Indices by Functional Category

Table 2 details the fatty acid composition and health-related lipid indices of milk across the two defined functional categories. Milk from biodiverse grazing systems was characterized by higher concentrations of oleic acid (30.0–33.4% vs. 20.7–35.2%) and lower atherogenic index (AI: 1.61–2.17 vs. 1.85–2.50) and thrombogenic index (TI: 2.31–2.70 vs. 2.30–3.10) compared to conventional systems. In contrast, the health promotion index (HPI) was consistently higher in the biodiverse category (0.46–0.67 vs. 0.40–0.54). These data reveal a systematic shift in the milk lipid profile, with biodiverse grazing systems yielding a higher proportion of unsaturated fatty acids relative to saturated fractions across all evaluated studies. However, substantial overlap in ranges for several fatty acids (oleic acid, ALA, EPA, MUFA, and n-6/n-3 ratio) indicates that the differences are not uniformly large across all individual fatty acids and that the most consistent effects are observed for the integrated indices (AI, TI, HPI) rather than for individual fatty acids.

### 3.2. Cheese Characteristics by Functional Category

Table 3 summarizes the chemical composition, lipid health indices, and bioactive profiles of cheeses produced under the two functional categories. Cheeses derived from biodiverse grazing systems (specifically organic cheese from Chiapas and Zebu-grazed cheese from Colima) exhibited a distinct phytochemical signature compared to conventional counterparts. While differences in health indices such as AI and TI were numerically narrow, a marked contrast was observed in the concentration of secondary metabolites. Specifically, biodiverse grazing cheeses showed substantially higher levels of tocopherols (127 vs. 77 mg 100 g^−1^) and monoterpenes (ranging from 460 to 475 vs. 111–126 ng kg^−1^). Furthermore, these cheeses were characterized by a consistent presence of long-chain n-3 fatty acids, such as EPA (4.7–5.4 mg 100 g^−1^), and lower total cholesterol levels compared to the conventional category.

### 3.3. Forage Nutritional Quality by Functional Category and Season

The study by Marroquín-Aguilar [29] evaluated the nutritional quality of four dominant grass species (*Cynodon nlemfuensis*, *Pennisetum merkeri*, *Brachiaria brizantha*, and *Panicum maximum* cv. Mombaza) across different seasons in Tecpatán, Chiapas. Crude protein (CP) content ranged from 6.7% to 12.9%, with higher values observed in *C. nlemfuensis* and *P. merkeri* during the rainy season. Neutral detergent fiber (NDF) ranged from 57.6% to 77.7%, and acid detergent fiber (ADF) ranged from 33.8% to 52.6%, with lower fiber content (indicating better digestibility) in *B. brizantha* and *C. nlemfuensis*. These nutritional parameters directly influence enteric methane emissions, as diets with higher fiber content and lower digestibility are associated with greater methane production per unit of dry matter intake. The seasonal variation in forage quality, with CP increasing and fiber decreasing during the rainy season, highlights the importance of timing in forage management for optimizing both animal performance and environmental outcomes.

### 3.4. Environmental Outcomes: Enteric Methane Emissions

Table 4 presents the comparative data for enteric methane (CH_4_) emissions and productive parameters between the functional categories. Based on the experimental results from Flores-Coello et al. [5], the biodiverse grazing system achieved an 18.2% reduction in total methane emissions (g d^−1^) compared to the conventional system. Notably, methane intensity, expressed per unit of dry matter intake (CH_4_ g kg^−1^ DMI), was 21.3% lower in the biodiverse category (*p* < 0.0001) than the conventional counterpart. This reduction in environmental impact was observed despite a significantly higher dry matter intake (+29.4%) in the biodiverse grazing cattle system, indicating a lower methane yield per unit of feed consumed in this system.

Also, estimates from Marroquín-Aguilar [29] using IPCC Tier 1 methodology showed that methane emissions varied by physiological state, with lactating cows producing the highest emissions (approximately 110–116 kg CH_4_ animal^−1^ year^−1^) and calves the lowest (approximately 36 kg CH_4_ animal^−1^ year^−1^). The effect of grass species on methane emissions was minimal (approximately 2 kg CH_4_ animal^−1^ year^−1^ difference between species), suggesting that animal factors (physiological state and body weight) are more significant determinants of enteric methane production than forage species alone in these systems.

### 3.5. Preclinical Metabolic Outcomes in a Murine Model

Table 5 details the physiological and metabolic responses of male C57BL/6 mice following a 14-week nutritional intervention. According to the findings of Cuchillo-Hilario et al. [30], the supplementation with lyophilized milk from the biodiverse grazing system significantly attenuated the negative effects of a high-fat diet (HFD). Mice in the HFD + Biodiverse milk group exhibited a final body weight (30.5 g) and fat mass percentage (19.2%) that were statistically similar to the Control diet group (29.4 g; 18.8%), effectively preventing the excessive adiposity observed in the HFD group (36.8 g and 29.8%, respectively). Furthermore, the biodiverse milk intervention nearly abrogated the development of hepatic steatosis and resulted in significantly smaller adipocyte sizes (1863 µm^2^) compared to both the HFD (3880 µm^2^) and the conventional milk (2257 µm^2^) treatments (*p* < 0.05). Metabolic flexibility was also influenced, as evidenced by the higher energy expenditure (VO_2_) and distinct respiratory exchange ratios (RER) in the milk-supplemented groups during the feeding phase, alongside improved glucose tolerance relative to the HFD control. These murine data provide supportive preclinical evidence for the metabolic benefits of biodiverse milk consumption, but they do not constitute direct evidence of human health effects.

## 4. Discussion

The central finding of this narrative synthesis is that functional traits: botanical diversity, concentrate dependence, and input intensity were consistently associated within the included studies with dairy product quality, environmental impact, and preclinical metabolic outcomes. Across six independent studies conducted over 20 years in three tropical regions of Mexico, systems classified as “biodiverse grazing” consistently exhibited more favorable profiles for atherogenic and thrombogenic indices, health promotion index, tocopherols, terpenes, methane emissions, and mouse metabolic outcomes compared to “conventional” systems. The consistency across studies is notable, particularly given the heterogeneity in cattle breeds (Gyr × Holstein, Brown Swiss × Brahman or pure Zebu), management (intensive silvopastoral, organic, traditional grazing), and geography (Yucatán, Chiapas, Colima). While inherent variables such as breed and parity may influence these parameters, the persistence of the functional trend across time and geography suggests a recurring association of the feeding system with the response variables within the included studies. Therefore, while we maintain a cautious stance aligned with recent calls to avoid overstating causal claims from observational data [15,16], these findings identify recurring associations between the independent and dependent variables examined.

Our findings are broadly consistent with literature demonstrating the benefits of biodiverse pastures for milk quality and environmental outcomes. In a study of nine Italian Alpine dairy farms, Pattono et al. [32] reported that the grazing season significantly affected the fatty acid and terpenoid profile of milk and cheese, with higher biodiversity associated with improved nutritional quality. Similarly, Radonjić et al. [33] demonstrated that mountain grassland diversity influences cow’s milk fatty acid composition, supporting the transferability of our functional classification concept to temperate systems. The inclusion of multispecies swards has been shown to enhance milk fatty acid profiles and oxidative stability [23,24]. The interactions between dietary regime and milk metabolomic profiles have been further corroborated by Connolly et al. [34], who showed that dietary regime impacts the metabolomic profile of bovine buttermilk and whole milk powder. Likewise, Timlin et al. [35] demonstrated that varying levels of pasture allowance to ruminants affect the nutritional quality and functionality of milk throughout the lactation period. These studies collectively support the mechanistic framework underlying our functional classification.

The relationship between forage diversity and milk fatty acid profile is well established in temperate systems [2,3]. Our synthesis extends these findings to tropical systems, where the diversity of plant secondary metabolites, such as condensed tannins, saponins, and flavonoids, exerts additional rumen-modulating effects [11]. For example, *Leucaena leucocephala* in biodiverse systems contains condensed tannins that can partially inhibit ruminal biohydrogenation, leading to higher concentrations of unsaturated fatty acids, particularly oleic acid, in milk [18]. In the organic and traditional grazing systems of Chiapas and Colima, the presence of diverse native legumes (*Fabaceae* family) likely produces similar effects. Lopera-Marín et al. [36,37] reported that silvopastoral systems with wild sunflower and yacon silage improved milk fatty acid profiles and production economics in Colombian dairy systems, providing additional support for the benefits of forage diversity in tropical contexts.

The magnitude of difference in AI and TI between biodiverse grazing and conventional systems was relatively small (e.g., AI 1.61 vs. 1.98). While statistically significant, the clinical relevance of such differences requires further investigation. However, the most pronounced contrasts were observed in the antioxidant and volatile profiles, where our results show a much larger margin of differentiation. Galina et al. [28] reported that cheese from grazing Zebu cattle contained nearly twice the tocopherol content of cheese from indoor-fed animals (127 vs. 77 mg 100 g^−1^). These lipid-soluble antioxidants serve a dual role: protecting polyunsaturated fatty acids from oxidation within the food matrix and potentially exerting independent anti-inflammatory effects in the consumer [38]. Similarly, Martini et al. [39] reported that organic cow milk contained higher concentrations of sterols, tocopherols, and bioactive fatty acids compared to conventional milk, corroborating our findings regarding the superior antioxidant profiles of biodiverse dairy products. Moreno-González et al. [40] further demonstrated that milk fatty acid dynamics in cows grazing conventional and multispecies pastures vary under different management systems, highlighting the importance of biodiversity in modulating milk composition.

The role of plant secondary metabolites in modulating ruminal biohydrogenation and enhancing milk bioactive compound content has been further elucidated by Delgadillo-Puga et al. [14], who demonstrated that supplementation with *Acacia farnesiana* pods, a forage rich in polyphenols and tannins, modified the fatty acid profile of goat milk and increased its antioxidant capacity. Similarly, Cuchillo et al. [4] reported that goat milk cheeses coming from animals grazing on Mexican rangelands exhibited significant antioxidant activity, attributed to bioactive polyphenols present in the forage species consumed by small ruminants. This supports the mechanistic framework proposed in our synthesis, wherein the botanical diversity of biodiverse grazing systems drives the transfer of phytochemicals into the milk matrix, contributing to both the superior antioxidant profiles and the metabolic benefits observed in our murine model.

Terpenes, which were 3 to 4-fold higher in grazed cheese (460–475 vs. 111–126 ng kg^−1^), are plant-derived secondary metabolites with documented anti-carcinogenic properties [11]. The transfer of these compounds suggests that the functional signature of biodiverse grazing systems is chemically stable and persists through processing, a prerequisite for practical application in functional foods. Recent advances in milk metabolomics have allowed researchers to discriminate feeding regimens based on metabolites [21]. Fleming et al. [22] emphasized that connecting grazing and milk metabolomes can enhance consumer health by identifying bioactive compounds that survive digestion. Our findings support this; the terpene profiles reported by Galina et al. [28] are a direct reflection of the forage intake (e.g., *Acacia* and *Prosopis*), and their persistence in the cheese matrix indicates they are not completely lost during fermentation or aging. Furthermore, Nidegger et al. [41] reported that calving season and seasonal diet composition influence butter color, firmness, and fatty acid profile in grazing systems, emphasizing the importance of considering temporal dynamics in dairy product quality assessment.

Regarding environmental efficiency, the 18.2% reduction in enteric methane emissions observed in biodiverse systems [5] is consistent with other studies of legume-based silvopastoral systems in the tropics [8,42]. This reduction is particularly significant as it identifies a potential convergence of nutritional and environmental benefits: the same botanical diversity that improves milk nutraceutical quality also appears to mitigate environmental impact. Several mechanisms explain these outcomes: first, higher quality forage improves digestibility and might reduce methane yield per unit of digested organic matter. Second, condensed tannins can suppress methanogenic archaea and protozoa populations in the rumen environment [17]. Third, the higher DMI in biodiverse systems (15.4–15.5 vs. 11.9–12.0 kg d^−1^) reduces methane production per kg DMI due to faster passage rates. The methane mitigation potential of biodiverse pastures has been corroborated by Loza et al. [43], who reported that grazing diverse pastures can predict rumen-derived methane. Likewise, Dwan et al. [20] demonstrated that increasing sward species diversity affects enteric methane emissions from dairy cows in rotational grazing systems, though the direction and magnitude of these effects depend on breed and management context. Soder and Brito [44] reviewed enteric methane emissions in grazing dairy systems, highlighting the potential for pasture management to mitigate emissions while maintaining productivity. Flores-Coronado et al. [45] conducted a greenhouse gas assessment comparing silvopastoral vs. monoculture cattle systems in a humid Mexican tropical region, finding significant reductions in net emissions from biodiverse systems. Similarly, Jaimes Cruz et al. [46] reported that enteric methane emissions from Holstein cows grazing Kikuyu grass in the high tropics varied by season, underscoring the importance of environmental context in methane mitigation strategies. Narváez-Herrera et al. [47] estimated enteric methane emissions in dairy cows grazing a silvopastoral system and a grass monoculture in the Amazonian foothills, finding lower emissions in the silvopastoral system.

The Marroquín-Aguilar [29] study adds an important dimension to this synthesis by quantifying forage nutritional quality and its relationship to methane emissions. The finding that grass species had minimal effect on methane emissions (approximately 2 kg CH_4_ animal^−1^ year^−1^ difference) compared to animal physiological state suggests that management strategies targeting animal factors (e.g., improved nutrition, reduced days to finish) may be more effective for methane mitigation than simply changing forage species. However, the higher crude protein content and lower fiber content observed in certain species (*C. nlemfuensis, B. brizantha*) during the rainy season indicate that seasonal forage management remains important for optimizing both animal performance and environmental outcomes. Gaviria et al. [42] reported that the nutritional quality, voluntary intake, and enteric methane emissions of diets based on Cayman grass and its associations with *Leucaena* shrub legumes varied significantly, supporting the importance of forage composition in methane mitigation. Likewise, Hernández et al. [48] demonstrated that milk yield and milk fatty acids from crossbred F1 dairy cows fed on tropical grasses varied with different levels of concentrate supplementation, highlighting the complexity of nutritional management effects on both productivity and milk composition.

It is important to note that the reduction in methane emissions per animal and per unit DMI was accompanied by a 20.7% reduction in milk yield (6.1 vs. 7.7 L d^−1^). However, when emissions were expressed per liter of milk produced, the biodiverse and conventional systems were comparable (approximately 60 g CH_4_ L^−1^ milk; [5]). This indicates that the environmental benefit of biodiverse systems lies primarily in methane intensity per unit of feed consumed rather than per unit of milk output. Whether this represents a trade-off or an acceptable compromise depends on the weighting of environmental versus productivity objectives in specific production contexts. Narváez-Herrera et al. [49] evaluated silvopastoral systems with native forage species and their impact on milk production and quality in the Amazonian foothills, reporting that biodiverse systems can maintain productivity while improving milk quality. In the same line, Lopera-Marín et al. [37] assessed milk production, principal composition, and economic performance of Holstein cows in a silvopastoral system with Mexican sunflower and yacon silage as partial concentrate replacement, finding that biodiverse systems can be economically viable while reducing concentrate dependence.

In terms of the impact of this functional classification on animal physiology and the potential impact on health, the murine model provides the strongest controlled evidence that compositional differences translate into differential metabolic outcomes. HFD mice fed biodiverse milk had final body weights not statistically different from those fed a low-fat control diet (30.5 vs. 29.4 g) in our model. Moreover, biodiverse milk completely abrogated hepatic steatosis, a marked effect given that HFD typically induces severe macrovesicular fat accumulation. The shift toward a lower respiratory exchange ratio (RER: 0.82 vs. 0.88 in HFD) and increased energy expenditure (VO_2_: ~4300 vs. ~3500 mL kg^−1^ h^−1^) indicates enhanced lipid oxidation and metabolic flexibility in rodents, suggesting that phytochemicals present in biodiverse milk may act as metabolic primers. Additionally, the hyperplastic (small adipocyte) phenotype observed in milk-fed mice (1863 µm^2^ vs. 3880 µm^2^ in HFD) suggests that milk consumption by mice promotes safer lipid storage. While milk intake per se improved adipose tissue function, the biodiverse milk produced the most favorable overall phenotype, including the lowest body weight and complete protection against hepatic steatosis. These findings justify future human trials comparing dairy products from biodiverse grazing versus conventional systems to corroborate these observations. Nakatani et al. [50] reported that bovine milk-derived extracellular vesicles ameliorated steatohepatitis in CDA-HFD-fed mice, suggesting that milk components beyond fatty acids and phytochemicals may contribute to the metabolic effects observed in our murine model. Wen et al. [51] conducted an exploratory lipidomic comparison of neutral and polar lipid profiles between mare and cow milk under a shared grazing environment, demonstrating that lipid composition varies across species and environmental conditions, which may influence the metabolic effects.

The role of milk composition in modulating obesity and metabolic dysfunction has been reviewed by Delgadillo-Puga and Cuchillo-Hilario [17], who highlighted the benefits of grazing/browsing semiarid rangeland feed resources and the transference of bioactivity and pro-healthy properties to goat milk and cheese. Their review supports the mechanistic framework underlying our findings, emphasizing the importance of plant secondary metabolites in conferring health benefits to dairy products from biodiverse systems. Redoy et al. [52] reported that supplementation of isoacids to lactating dairy cows fed low- or high-forage diets affected performance, digestibility, and milk fatty acid profile, further illustrating the complexity of interactions between dietary components and milk composition.

## 5. Limitations and Future Directions

Several limitations must be considered while interpreting the conclusions of this synthesis. The classification into functional categories, although mechanistically grounded, relies on thresholds (e.g., >15 species for high diversity) that require further calibration. Different thresholds could alter the assignment of borderline systems, and formal independent external validation beyond our research group is lacking. The thresholds from this narrative review were derived from the distribution of plant species richness observed across the included studies, but they were not empirically validated through sensitivity analyses or independent datasets. Future work should systematically evaluate alternative threshold values using larger and more diverse datasets.

Geographically, only six studies from a single country (Mexico) were included; thus, the applicability of these functional categories to other tropical regions with distinct forage ecologies, such as Brazil, East Africa, or Southeast Asia, remains to be established. Cross-regional comparisons involving tropical systems from different continents would be valuable to test the generalizability of our classification. Heterogeneity in study design, varying outcome measures, diverse breeds, and disparate analytical platforms precluded a formal meta-analysis. Observed associations may be confounded by unmeasured factors such as farmer management circumstances, soil quality, season of the year, altitude or water-stress impact on forage secondary metabolites. We did not systematically measure polyphenols, condensed tannins, or rumen microbiota across all studies, leaving the precise causal relationships partially unconfirmed.

The methane emission estimates from Marroquín-Aguilar [29] were based on IPCC Tier 1 methodology using default emission factors, which may not fully capture the specific conditions of tropical grazing systems in Mexico. Direct measurement of methane emissions (e.g., using SF_6_ tracer or GreenFeed systems) would provide more accurate estimates. The direct measurement data from Flores-Coello et al. [5] provide more reliable estimates, but these were limited to a single location (Yucatán) and breed type, limiting generalizability. The murine model, while offering high mechanistic control, does not replicate the full complexity of human metabolism; therefore, extrapolation to human health requires stringent controlled dietary trials.

The range of fatty acid values for ALA, EPA, and other individual fatty acids showed substantial overlap between biodiverse and conventional systems (Table 2), indicating that the most consistent differences were observed for the integrated health indices (AI, TI, HPI) rather than for individual fatty acids. This suggests that the functional classification may be more useful for predicting composite lipid quality outcomes than for predicting specific fatty acid concentrations, though future studies with larger sample sizes may reveal more consistent patterns for individual fatty acids.

The narrative synthesis approach, while appropriate given the heterogeneity of the source studies, does not provide quantitative effect estimates or statistical measures of heterogeneity. The qualitative comparisons presented in the Results should be interpreted as descriptive patterns rather than statistically tested differences between functional categories. Future formal meta-analyses should become feasible with more homogeneous datasets and would provide more robust quantitative evidence. However, at present, the evidence supports internal consistency within the included studies rather than empirical validation of the proposed thresholds.

Addressing these limitations will require a coordinated research agenda spanning multiple disciplines. Double-blind human intervention trials comparing dairy from biodiverse versus conventional systems represent the highest priority, as they would confirm whether the compositional differences observed in our preclinical model translate into clinically meaningful health outcomes in human consumers. Several recent studies have demonstrated the importance of diet composition and management factors in determining milk quality. De La Torre-Santos et al. [53] showed that the mode of grass supply to dairy cows impacts the fatty acid and antioxidant profile of milk. Hernández et al. [48] demonstrated that milk yield and fatty acid profiles from crossbred F1 dairy cows fed tropical grasses varied with concentrate supplementation levels. These studies highlight the need for integrated approaches that consider multiple management factors simultaneously.

Concurrently, high-resolution metabolomics and lipidomics should be applied systematically to milk and cheese samples, enabling identification of the specific bioactive phytochemicals that survive processing and resist gastrointestinal digestion. Such analytical efforts would be greatly facilitated by standardized reporting protocols for botanical diversity, concentrate dependence, and input intensity across studies, a prerequisite for future robust meta-analyses capable of detecting subtle but important effects obscured by current methodological heterogeneity. The application of advanced analytical techniques has been demonstrated by Rocchetti et al. [21] and Frizzarin et al. [54], who used metabolomics to discriminate different cows’ feeding regimens. Connolly et al. [34] further showed that dietary regime impacts the metabolomic profile of bovine buttermilk and whole milk powder, providing a methodological template for future studies.

Mechanistic investigations are paramount to elucidate how biodiverse grazing milk induces hyperplastic adipose remodeling and to determine whether specific fatty acids, lipokines, or phytochemical-derived metabolites mediate this effect. The potential role of milk-derived extracellular vesicles in metabolic modulation, as demonstrated by Nakatani et al. [50], represents a particularly promising avenue for future research. These studies should be complemented by comprehensive life cycle assessments and economic feasibility analyses to quantify the net environmental and financial benefits of transitioning to biodiverse grazing systems. International cross-regional comparisons are similarly critical to test the generalizability of our functional classification beyond Mexico, while direct measurement of methane emissions in tropical grazing systems, using tracer gas or open-circuit respirometry techniques, would validate the IPCC-based estimates that currently underpin our environmental conclusions. The studies by Flores-Coronado et al. [45], Jaimes Cruz et al. [46], and Narváez-Herrera et al. [47] provide examples of such direct measurements in tropical systems that could serve as models for future validation studies.

Exploring the persistence of bioactive compounds through human digestion and integrating “One Health” metrics into research designs will be essential to connect farm-level management decisions to human and planetary health outcomes [25]. Collectively, these research directions will move the field from descriptive associations toward actionable evidence for public health policy and sustainable livestock practices.

## 6. Conclusions

A functional classification system based on botanical diversity, concentrate dependence, and input intensity provides a more biologically informative framework than traditional administrative labels, though it requires further external validation. Across six independent studies in tropical Mexico, biodiverse grazing systems (including intensive silvopastoral, organic, and traditional grazing) were consistently associated with: (i) improved lipid quality markers, such as lower atherogenic and thrombogenic indices and higher health promotion indices; (ii) a superior nutraceutical profile, characterized by approximately doubled tocopherol content and three-fold higher terpene concentrations; (iii) a reduced environmental footprint, evidenced by an 18.2% reduction in enteric methane emissions per animal and 21.3% reduction per unit dry matter intake; and (iv) markedly more favorable preclinical metabolic outcomes in a murine model, most notably the prevention of diet-induced obesity and the near-complete mitigation of hepatic steatosis.

From a One Health perspective, biodiverse grazing systems offer a unique synergy between environmental sustainability (through reduced methane emissions, elimination of agrochemicals, and potential carbon sequestration) and preclinical health benefits (as evidenced by the murine model). The included studies suggest that milk is not a static animal-derived product; it is a biological output that carries the functional signature of the landscape’s ecological and animal physiology complexity. The challenge for veterinary, animal, and public health sciences is to move beyond simplistic labels and rigorously test whether managing livestock feeding systems as deliberate nutritional interventions can simultaneously improve human, animal, and planetary health. While the functional classification framework presented here shows promise, its ultimate utility will depend on external validation, empirical calibration of thresholds, and translation of preclinical findings to human health outcomes through well-designed intervention trials.

## Figures and Tables

**Table 1:** Functional classification of cattle treatment feeding systems in tropical Mexico.

Functional Category	Studies and Treatments Included	Location	Key Traits	Outcomes Measured
Biodiverse grazing systems	Flores-Coello et al. [5]—SPS; Cuchillo-Hilario et al. [30]—SPS; Delgadillo-Puga et al. [9]—Organic; Cruz-Morales [10]—Organic; Galina et al. [28]—Grazing; Marroquín-Aguilar [29]—Traditional grazing	Yucatán (Dzununcán); Chiapas (Tecpatán); Colima (Comala).	High botanical complexity (>15 species); use of nitrogen-fixing legumes (e.g., *L. leucocephala*). No synthetic inputs (herbicides/fertilizers); manual milking; concentrate <25% of DMI.	Milk/cheese FA profiles, AI/TI/HPI indices, antioxidants (tocopherols, terpenes), somatic cell counts (SCC), enteric CH_4_ emissions, forage nutritional quality
Conventional/Monoculture systems	Flores-Coello et al. [5]—MS; Cuchillo-Hilario et al. [30]—MS; Galina et al. [28]—Indoor; Delgadillo-Puga et al. [9]—Conventional; Cruz-Morales [10]—Conventional; Marroquín-Aguilar [29]—Monoculture pastures	Yucatán (Xmatkuil); Chiapas (Tecpatán); Colima (Comala).	Low botanical diversity (grass monocultures, 3 to 5 species). Use of agrochemicals (urea and herbicides). High concentrate dependence (40–45% of DMI); intensive stocking or confinement.	FA profiles, higher AI/TI indices, higher SCC, higher enteric CH_4_ per kg of DMI, mouse obesity and steatosis outcomes *, forage fiber and protein content

DMI = dry matter intake; FA = fatty acids; AI = atherogenic index; TI = thrombogenic index; HPI = health promotion index; SCC = somatic cell count; CH_4_ = methane. * Mouse metabolic outcomes (weight gain, glucose tolerance, and liver steatosis) were specifically evaluated using milk from Yucatán systems [30]. SPS (Silvopastoral System); MS (Monoculture System).

**Table 2:** Representative ranges of milk fatty acids and cardiovascular health indices across biodiverse and conventional dairy systems in tropical Mexico (Yucatán and Chiapas).

Parameter	Biodiverse Milk	Conventional Milk	Reference
Oleic acid (C18:1 cis-9, %)	30.0–33.4	20.7–35.2	[9,30]
ALA (C18:3 n-3, %)	0.55–0.67	0.51–0.70	[9,30]
EPA (C20:5 n-3, %)	0.04–0.13	0.04–0.15	[9,30]
SFA (%)	57.5–63.2	58.7–73.3	[9,30]
MUFA (%)	32.3–35.6	22.5–37.0	[9,30]
n-6/n-3 ratio	2.0–9.4	1.0–9.6	[9,30]
Atherogenic Index (AI)	1.61–2.17	1.85–2.50	[9,30]
Thrombogenic Index (TI)	2.31–2.70	2.30–3.10	[9,30]
Health Promotion Index (HPI)	0.46–0.67	0.40–0.54	[9,30]

AI and TI values are considered more favorable at lower levels, whereas HPI is more favorable at higher levels. Reported ranges reflect seasonal and regional variation across dairy production systems in tropical Mexico, including organic systems in Chiapas and grazing-based/conventional systems in Yucatán. Cuchillo-Hilario et al. [25] in Yucatán, Mexico; Delgadillo-Puga et al. [9] in Chiapas, Mexico.

**Table 3:** Fatty acid profile, health-related lipid indices, and nutraceutical compounds of cheeses from biodiverse and conventional dairy systems in tropical Mexico (Colima and Chiapas).

Parameter	Biodiverse Cheeses	Conventional Cheeses	Reference
Oleic acid (C18:1 cis-9) (mg 100 g^−1^)	1160–1178	1198–1310	[10,28]
ALA (C18:3 n-3) (mg 100 g^−1^)	41–44	37–39	[28]
EPA (C20:5 n-3) (mg 100 g^−1^)	4.7–5.4	3.6–4.3	[28]
SFA (mg 100 g^−1^)	2490–2960	2720–3350	[10,28]
MUFA (mg 100 g^−1^)	1220–1290	1180–1390	[10,28]
PUFA (mg 100 g^−1^)	150–270	170–250	[10,28]
n-6/n-3 ratio	1.81–3.92	1.97–3.40	[10,28]
Atherogenic Index (AI)	1.90–2.03	1.89	[10]
Thrombogenic Index (TI)	2.50–2.63	2.53	[10]
Health Promotion Index (HPI)	0.51–0.53	0.53	[10]
Tocopherols (mg 100 g^−1^ DM)	127	77	[28]
Monoterpenes (ng kg^−1^)	460–475	111–126	[28]
Sesquiterpenes (ng kg^−1^)	520–1314	210–935	[28]
Cholesterol (mg 100 g^−1^)	70.5	79.1	[28]

Headers “Biodiverse cheese” and “Conventional cheese” refer to cheeses derived from biodiverse and conventional dairy production systems, respectively. Lower AI and TI values and higher HPI values are considered nutritionally favorable. Cruz-Morales [10] in Chiapas, Mexico; Galina et al. [28] in Colima, Mexico.

**Table 4:** Comparative enteric methane emissions and productive parameters of cattle under Biodiverse and Conventional production systems in Yucatán, Mexico. Direct measurement data from Flores-Coello et al. [5] are presented in the first four rows; IPCC-based estimates from Marroquín-Aguilar [29] are presented in the final row.

Parameter	Biodiverse	Conventional	% of Difference	*p*-Value
Total CH_4_ (g d^−1^) Direct measurement	376 (261–491)	460 (264–663)	–18.2%	<0.05
CH_4_ Intensity (g kg^−1^ DMI) Direct measurement	24.0 ± 3.5	30.5 ± 5.8	–21.3%	<0.0001
Dry Matter Intake (kg d^−1^) Direct measurement	15.4–15.5	11.9–12.0	+29.4%	<0.05
Milk Yield (L d^−1^) Direct measurement	6.1 ± 1.8	7.7 ± 2.1	–20.7%	<0.0001
Total CH_4_ (g d^−1^) IPCC Tier 1 estimates	376 (261–491)	460 (264–663)	–18.2%	<0.05

Data sources: Direct measurement data from [5]; IPCC-based estimates from Marroquín-Aguilar [29].

**Table 5:** Metabolic and physiological outcomes in C57BL/6 mice fed a high-fat diet supplemented with milk from biodiverse and conventional tropical dairy systems (14-week intervention).

Outcome	Control Diet (CD, 7% Fat)	High-Fat Diet (HFD, 21% Fat)	HFD+ Biodiverse Dairy Systems	HFD+ Conventional Dairy Systems
Final body weight (g)	29.4 ± 1.5 ^c^	36.8 ± 1.8 ^a^	30.5 ± 1.2 ^bc^	33.8 ± 1.4 ^abc^
Fat mass (%)	18.8 ± 4.5 ^b^	29.8 ± 2.5 ^a^	19.2 ± 3.8 ^b^	20.0 ± 2.8 ^ab^
Lean mass (%)	77.9 ± 3.6 ^a^	68.1 ± 1.8 ^b^	75.0 ± 4.5 ^ab^	76.3 ± 2.5 ^ab^
Hepatic steatosis	None	Severe macrovesicular	Nearly abrogated	Significantly reduced
Adipocyte size (SAT, µm^2^)	1193 ± 315 ^d^	3880 ± 950 ^a^	1863 ± 410 ^c^	2257 ± 520 ^b^
Respiratory exchange ratio (RER)	0.99 ± 0.01 ^a^	0.88 ± 0.02 ^b^	0.82 ± 0.01 ^c^	0.83 ± 0.01 ^c^
Energy expenditure (VO_2_, mL kg^−1^ h^−1^) *	~3700 ^c^	~3500 ^d^	~4300 ^a^	~3900 ^b^
Intraperitoneal glucose tolerance test (IPGTT AUC)	26,858 ± 2100 ^b^	45,248 ± 3500 ^a^	32,616 ± 2800 ^ab^	35,645 ± 3200 ^ab^

* Values for energy expenditure (VO_2_) represent the feeding phase, where the metabolic impact of milk was most evident. Different superscript letters within rows indicate significant differences (*p* < 0.05). Data adapted from Cuchillo-Hilario et al. [30].

## Data Availability

The original contributions presented in this study are included in the article/Supplementary Material. Further inquiries can be directed to the corresponding author.

## Supplementary Materials

The following supporting information can be downloaded at: https://www.mdpi.com/article/10.3390/vetsci13090988/s1, Table S1: Characteristics of the six source studies included in the narrative synthesis.

## Abbreviations

The following abbreviations are used in this manuscript:

AI	Atherogenic Index
ALA	α-Linolenic acid (C18:3 n-3)
CD	Control Diet (7% fat in mouse study)
CH_4_	Methane
CLA	Conjugated Linoleic Acid
CONACYT	Consejo Nacional de Ciencia y Tecnología (Mexico)
CP	Crude Protein
DMI	Dry Matter Intake
EPA	Eicosapentaenoic acid (C20:5 n-3)
FA	Fatty Acids
HFD	High-Fat Diet (21% fat in mouse study)
HOMA-IR	Homeostatic Model Assessment of Insulin Resistance
HPI	Health Promotion Index
IL-6	Interleukin-6
LDL/HDL	Low-Density Lipoprotein/High-Density Lipoprotein
MS	Monoculture pasture system (or Monoculture System)
MUFA	Monounsaturated Fatty Acids
NDF	Neutral Detergent Fiber
PPARγ	Peroxisome Proliferator-Activated Receptor Gamma
PUFA	Polyunsaturated Fatty Acids
RER	Respiratory Exchange Ratio
SCC	Somatic Cell Count
SFA	Saturated Fatty Acids
SPS	Intensive Silvopastoral System
TI	Thrombogenic Index
TNF-α	Tumor Necrosis Factor Alpha
