# Supplementary material for: Functional Classification of Tropical Cattle Feeding Systems Reveals Consistent Associations with Dairy Lipid Quality, Enteric Methane Emissions, and Preclinical Metabolic Outcomes: A Multi-Study Narrative Synthesis
Source: Vet Sci. 2026 Sep 18;13(9):988. doi: 10.3390/vetsci13090988 (PMC13612096; doi:10.3390/vetsci13090988)
Supplement: Supplementary file 1 [file vetsci-13-00988-s001.zip › vetsci-4539024-supplementary.pdf]

Table S1. Characteristics of the six source studies included in the narrative synthesis

Citation	Galina et al. [28]	Cruz-Morales, [10]	Delgadillo-Puga et al. [9]	Marroquín-Aguilar [29]	Flores-Coello et al. [5]	Cuchillo-Hilario et al. [30]
Year(s) of data collection	2004 (spring and summer)	November 2010 - April 2011	January - June 2009	April 2012 - March 2013	April - May 2016 and October 2016	April to August 2023
Location	Colima: Comala Querétaro: Cerro Prieto	Chiapas: Tecpatán (Ejido Emiliano Zapata)	Chiapas: Grijalva, Pomarroza and Malpaso	Chiapas: Ejido Emiliano Zapata, Tecpatán	Yucatán: Xmatkuil Dzununcan	Yucatán: Xmatkuil Dzununcan Catmis, Tzucacab
Number of farms/units [1]	2 farms	2 production units from "La Pomarrosa" group	75 farms	1 ejido with 25 grazing units	2 commercial farms	3 farms (1 per system: SPS, MS, NVS)
Number of animals	14 Zebu cows; 60 Alpine crossbred goats	900 cows total (444 organic, 456 conventional), distributed in three communities.	750 cows total (303 organic, 447 conventional), distributed in three communities.	264 cows total (132 organic, 132 conventional), in a community or ejido Emiliano Zapata (SPR Pomarroza) .	60 lactating Gyr × Holstein crossbred cows (40 MS, 20 SPS)	30 lactating dual-purpose cows (10 per system)
Breed	Zebu cows (Colima); Alpine crossbred goats (Querétaro)	Zebu-Holstein, Zebu-American Swiss, and American Swiss cross-bred cows	Cross-breed cows (Zebu-Holstein, Zebu-American Swiss, American Swiss)	Zebu-Holstein, Zebu-American Swiss, and American Swiss cross-bred cows	Gyr × Holstein crossbred (Bos indicus × Bos taurus)	Gyr × Holstein crosses (SPS, MS); Brown Swiss × Brahman crosses (NVS)
Study design	Comparative experimental (grazing vs. indoor fed)	Observational/descriptive (organic cheese characterization)	Comparative cross-sectional (organic vs. conventional farming systems)	Observational/descriptive (forage analysis and methane estimation)	Comparative experimental (intensive silvopastoral vs. monoculture)	Experimental (milk from biodiverse vs. conventional systems fed to mice)
Experimental unit	Individual animals; pooled milk per group	Cheese samples	Milk samples (bulk tank)	Forage plots (0.25–2 m²) and herd-level emissions	Paddocks (0.25–0.35 ha) and cows	Mice (n = 6 per group) 5 groups.

Feeding systems compared	Grazing (shrub land with tropical legume forest supplement vs. indoor fed)	Organic cheese (from organic production system)	Organic vs. conventional farming systems	Traditional grazing (four grass species evaluated)	Intensive silvopastoral system (ISPS) vs. monoculture system (MS)	Silvopastoral (SPS) vs. Monoculture (MS) vs. Native Vegetation (NVS)
Key traits of biodiverse system	Grazing on 16 ha of tropical legume forest with grasses supplemented	Organic production; traditional agrosilvopastoral management	Organic farming; traditional agrosilvopastoral management; no synthetic inputs	Traditional grazing: *Brachiaria brizantha*, *Megathyrsus maximus* cv. Mombasa, *Brachiaria decumbens*, *Cynodon nlemfuensis*, Cenchrus purpureus, cubano (CTC-115; *Pennisetum purpureum x Pennisetum glaucum*). Open pasture (ha): Traditional grazing system 23.7 ha. Pasture with scattered trees (ha) in traditional grazing system 10.35 ha average per farm. Agrosilvopastoral management; no synthetic inputs	ISPS with >15 plant species; *L. leucocephala*; no synthetic inputs; lower concentrate dependence	Silvopastoral system with higher botanical diversity; *L. leucocephala*; trees for shade
Key traits of conventional system	Indoor fed: corn silage, King grass and supplementation	Not applicable (only organic cheese evaluated)	Conventional farming; use of agrochemicals and external inputs	Monoculture pastures Open pasture (ha): Conventional system 23.5 ha average per farm. Pasture with scattered trees (ha) in conventional system, 2.7 ha average per farm. Use of agrochemicals and external inputs.	Monoculture system (MS) with lower botanical diversity; higher concentrate dependence; agrochemicals	Monoculture system; *C. nlemfuensis*; agrochemicals; higher concentrate dependence
Outcomes evaluated	Cheese quality, chemical	Fatty acid profile, atherogenic index,	Fatty acid profile, health and risk	Forage nutritional quality (CP, NDF, ADF); methane	Enteric methane	Milk composition; metabolic outcomes

	composition, fatty acid profile, cholesterol, tocopherols, terpenes	thrombogenic index, health promotion index of organic cheese	indices (HPI, TI), physicochemical composition, somatic cell counts	emissions (IPCC Tier 1 estimates)	emissions (sniffer methodology), dry matter yield, nutrient intake, milk yield, body condition score	in C57BL/6 mice (body weight, fat mass, glucose tolerance, hepatic steatosis, adipocyte size)
Analytical methodology	Chemical analysis of cheese; GC/MS for terpenes; silver-ion HPLC for CLA; AOAC methods	Lipid analysis by GC; physicochemical analysis per NMX standards	Fatty acid analysis by GC; HPI and TI calculation	Forage chemical analysis (CP, NDF, ADF per AOAC and Van Soest); IPCC Tier 1 methane estimation	Sniffer methodology for CH_4; AOAC and Van Soest methods for forage analysis	Murine metabolic phenotyping (EchoMRI, CLAMS, ipGTT); milk composition analysis
Source type	Peer-reviewed journal article	Bachelor's thesis	Peer-reviewed journal article	Bachelor's thesis	Peer-reviewed journal article	Peer-reviewed journal article

SPS = Silvopastoral System; MS = Monoculture System; NVS = Native Vegetation System; ISPS = Intensive Silvopastoral System;; OM = Organic Milk; CM = Conventional Milk; HPI = Health Promotion Index; TI = Thrombogenic Index; AI = Atherogenic Index; SCC = Somatic Cell Count; CP = Crude Protein; NDF = Neutral Detergent Fiber; ADF = Acid Detergent Fiber; GC = Gas Chromatography; GC/MS = Gas Chromatography-Mass Spectrometry; HPLC = High-Performance Liquid Chromatography; CLA = Conjugated Linoleic Acid; AOAC = Association of Official Analytical Chemists; NMX = Norma Mexicana (Mexican Standard); IPCC = Intergovernmental Panel on Climate Change; EchoMRI = Echo Magnetic Resonance Imaging; CLAMS = Comprehensive Lab Animal Monitoring System; ipGTT = Intraperitoneal Glucose Tolerance Test. [1] Total farms/units summed across all six studies: 2 + 2 + 75 + 25 + 2 + 3 = 109.
